# Biomechanical comparison of lumbar rotational manipulations in two different positions using force measurement and finite element analysis

**DOI:** 10.1186/s12998-026-00683-7

**Published:** 2026-10-05

**Authors:** Jiasi Shen, Xilin Gao, Hongen Zheng, Xinyi Zhang, Zhen Li, Yue Fan, Wei Zhang, Ping Wang

**Affiliations:** 1 First Teaching Hospital of Tianjin University of Traditional Chinese Medicine, Tianjin, 300381 China; 2 National Clinical Research Center for Chinese Medicine, Tianjin, 300381 China; 3 Xinjiang Production and Construction Corps Hospital, Xinjiang, 830002 China; 4 The Second Affiliated Hospital of Shihezi University School of Medicine Urumqi, Xinjiang, 830002 China

**Keywords:** Force measurement, Finite element analysis, Lumbar rotational manipulation, Lateral decubitus lumbar manipulation, Sitting lumbar manipulation, Biomechanics of spinal manipulation

## Abstract

**Purpose:**

To investigate the biomechanical characteristics of lateral decubitus and sitting lumbar rotational manipulations (LRM) by combining experimental force measurements with finite element analysis (FEA), with a particular focus on regional stress and displacement distributions and areas of stress concentration that may have relevance for future clinical and safety studies.

**Methods:**

Twenty healthy volunteers were randomly assigned to receive either lateral decubitus or sitting LRM. Practitioner-generated forces were recorded using instrumented gloves, and preliminary cluster analysis was performed to explore distinct operational patterns. The measured loading parameters were then used to define the loading conditions in a validated lumbar finite element model to compare stress and displacement distributions in the intervertebral discs and facet cartilage across the two techniques and five clusters.

**Results:**

Preliminary cluster analysis identified two clusters for the lateral decubitus technique (shoulder-hip coordinated and hip-dominant) and three for the sitting technique (shoulder-waist coordinated, shoulder-dominant, and gentle-loading). No significant left-right differences in practitioner-generated forces were observed for the lateral decubitus technique (all *P* > 0.05). For the sitting technique, the force applied at the shoulder was lower on the left than on the right (62.56 ± 15.58 vs. 77.70 ± 7.72 N; *t* = -10.088, *P* < 0.001), whereas the force applied at the waist was higher on the left than on the right [32.80 (27.75–38.20) vs. 26.06 (22.66–33.54) N; *Z* = 2.129, *P* = 0.042]. FEA showed distinct mechanical patterns between the two techniques. Across the two lateral decubitus clusters, the maximum intervertebral disc stress ranged from 7.87 to 13.40 MPa and the maximum deformation from 7.22 to 12.31 mm, with the main mechanical effects concentrated at L3/4–L4/5. Across the three sitting clusters, the maximum disc stress ranged from 5.87 to 8.09 MPa and the maximum deformation from 11.68 to 16.44 mm, with greater displacement mainly involving L1/2–L2/3. A similar contrast was observed in the facet cartilage, with the lateral decubitus technique generally producing higher peak stress but lower deformation than the sitting technique.

**Conclusion:**

Lateral decubitus and sitting LRM produced distinct regional biomechanical patterns. Lateral decubitus manipulation was characterized by a higher-stress/lower-displacement profile predominantly involving the lower lumbar spine, whereas sitting manipulation showed a lower-stress/higher-displacement profile with greater involvement of the upper lumbar spine. These findings provide a quantitative biomechanical basis for further investigation of technique-specific loading characteristics, tissue safety, and individualized clinical application.

**Clinical trial number:**

Not applicable.

## Introduction

Lumbar rotational manipulation (LRM) is a widely practiced non-surgical intervention for lumbar disc herniation (LDH), facet joint disorders, and other forms of acute and chronic low back pain. Its clinical efficacy has been well established, with documented benefits in alleviating nerve root compression and reducing soft tissue adhesions [1–3]. Among the various techniques, lateral decubitus and sitting LRMs are the most frequently used. These approaches operate through distinct biomechanical mechanisms: axial pelvic loading in the lateral decubitus position and spinal rotational leverage in the sitting position, thereby producing therapeutic effects in different regions of the lumbar spine [4, 5]. Nevertheless, the biomechanical transmission and stress distribution within key lumbar structures, such as intervertebral discs and facet cartilage, remain poorly understood. In practice, manipulative procedures largely depend on the operator’s personal experience, which limits the reproducibility of clinical outcomes and may expose patients to non-negligible risks [6–9].

Conventional spinal biomechanics research has largely relied on cadaveric specimens or in vivo models, which, although representative of human anatomy, are constrained by ethical and practical limitations [10]. Finite element analysis (FEA), which combines three-dimensional imaging with mathematical modeling, enables the simulation of lumbar morphology and mechanical responses in a virtual environment, offering an effective approach to elucidate the biomechanical mechanisms underlying spinal manipulation [11, 12]. Nonetheless, most existing FEA studies have concentrated on single manipulation techniques, with limited systematic comparisons across different procedures. Furthermore, these studies often neglect real clinical operating conditions and fail to incorporate experimentally measured practitioner-generated forces as boundary inputs for simulation, thereby reducing the predictive reliability of the models.

Building on these considerations, the present study employed an integrated in vivo and in silico approach combining experimental data with computational modeling. Practitioner-generated forces were recorded during manipulation using an intelligent force-sensing glove and used to define loading conditions in a self-developed three-dimensional finite element model of the lumbar spine, thereby enabling simulation of the immediate biomechanical effects of the procedures. The primary aims were to characterize kinematic and dynamic variables by combining measured practitioner-generated forces with FEA-derived tissue responses, systematically compare the mechanical responses elicited in intervertebral discs and facet cartilage by lateral decubitus and sitting techniques and their respective clusters, and identify regional differences in stress concentration and displacement. These findings are intended to advance understanding of the biomechanical mechanisms of spinal manipulation and to provide a theoretical foundation for the development of standardized, evidence-based clinical protocols.

## Materials and methods

### Acquisition of practitioner-generated forces for the two rotational manipulations

#### Data sources

From June 2024 to June 2025, a total of 20 healthy volunteers (age 20–30 years, body mass index [BMI] 18–24 kg/m²) without a history of spinal disease were recruited. Participants were randomly assigned to either the lateral decubitus lumbar rotational manipulation group or the sitting lumbar rotational manipulation group (*n* = 10 per group). All procedures were performed by an experienced senior clinician using an intelligent force-sensing glove to record practitioner-generated forces during manipulation. Each participant received three manipulations on each side during the same experimental session.

#### Instrumentation

**Fig. 1:**
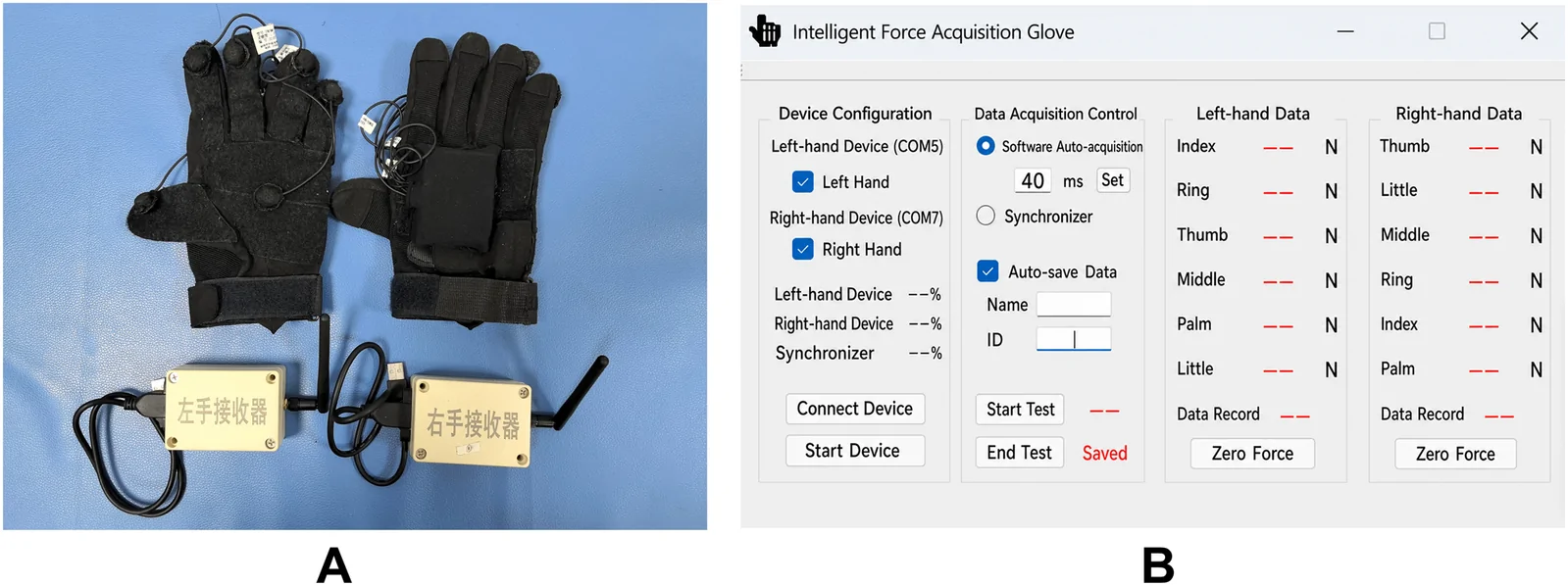
Intelligent force-sensing glove system. (**A**) Pressure sensors, hardware circuit module, and wireless transmission unit; (**B**) Dedicated data acquisition software

The intelligent force-sensing glove was jointly developed by the Orthopedic Technology Laboratory of the China Academy of Chinese Medical Sciences and the Beijing Institute of Technology. The system comprises pressure sensors, hardware circuit modules, wireless transmission units (Fig. 1A), and dedicated data acquisition software (Fig. 1B). Thin-film pressure sensors adhered to the glove surface capture mechanical signals, which are wirelessly transmitted to a computer for real-time recording and extraction of force data.

#### Experimental procedure

**Fig. 2:**
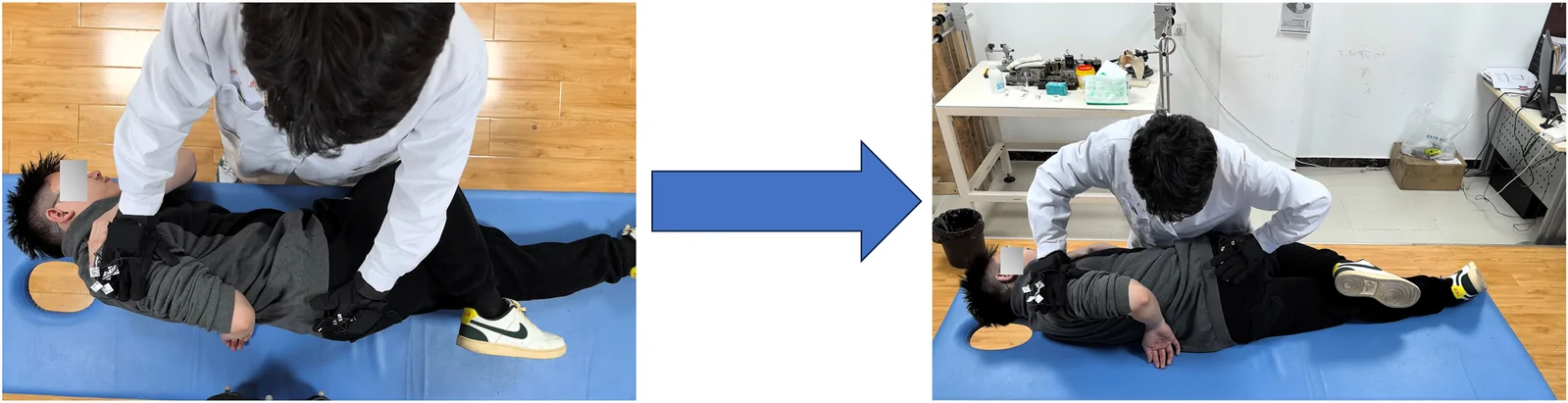
Illustration of the lateral decubitus lumbar rotational manipulation

**Fig. 3:**
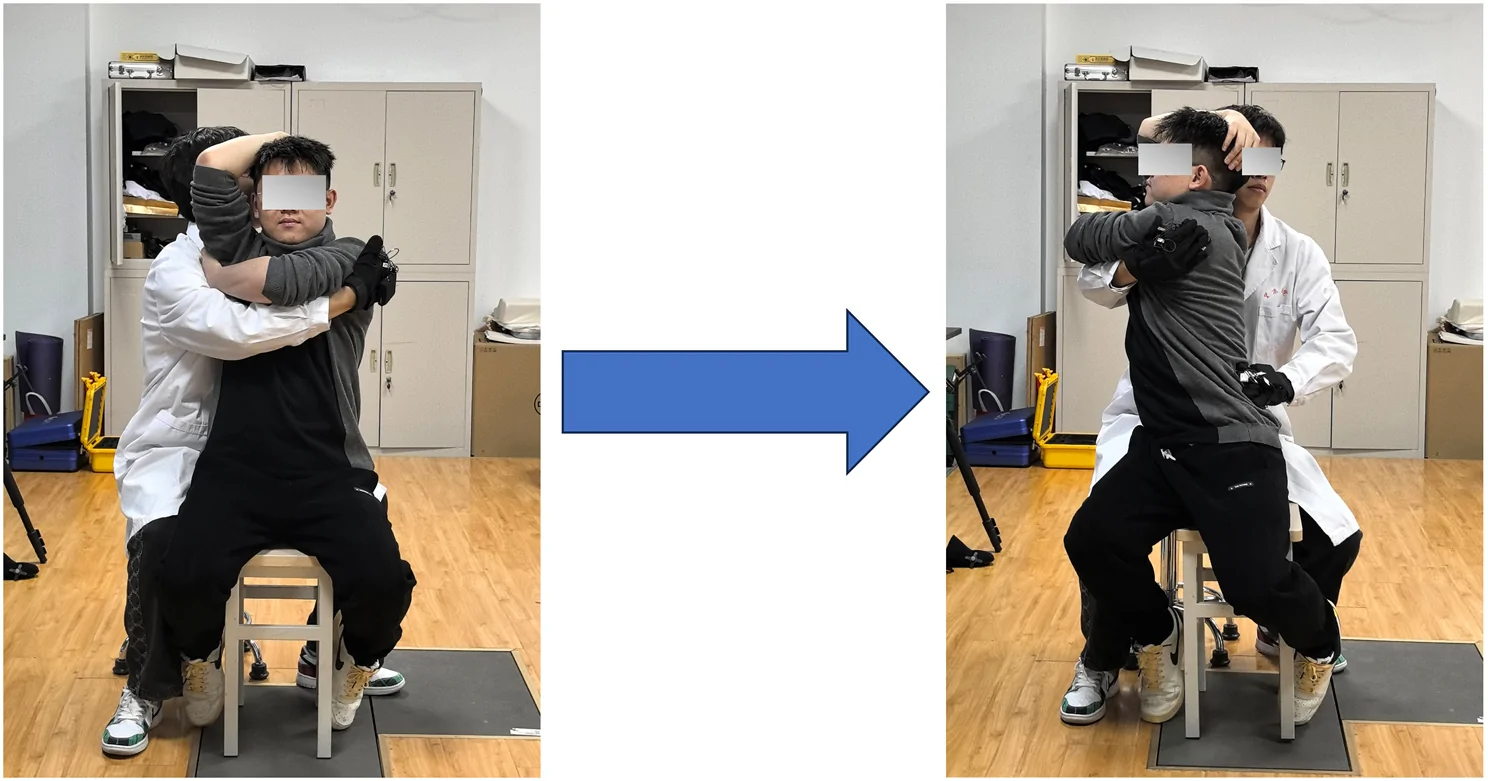
Illustration of the sitting lumbar rotational manipulation

1. Lateral decubitus lumbar rotational manipulation: Using the right side as an example, the subject was positioned on the treatment table in a left lateral decubitus posture, with the left leg extended and the right leg flexed at the hip and knee, resting atop the left leg. The left arm was flexed at 90° and held against the chest, while the right arm was extended posteriorly along the back. The operator, standing on the ventral side of the subject and wearing an intelligent force-sensing glove, placed the left hand on the posterior aspect of the right hip and the right hand on the anterior aspect of the right shoulder. Rotational movements were then performed with the spine serving as the axis. After achieving joint elastic fixation, the operator delivered a synchronized, short oblique thrust with both hands (Fig. 2). The contralateral lateral decubitus manipulation was performed in the reverse manner.
2. Sitting lumbar rotational manipulation: Using the right side as an example, the subject was seated astride a square wooden stool with both legs naturally apart, the right hand resting on the top of the head, and the left hand positioned beneath the right axilla across the chest. The operator, seated behind the subject and wearing intelligent force-sensing gloves, placed the left thumb pad on the right side of the L4 spinous process while the right arm passed through the subject’s right axilla to rest on the left shoulder. The subject’s lumbar spine was gently rotated to the right, during which the operator’s left thumb gradually applied a directed force to the spinous process toward the left. Once a sense of joint locking was achieved, the operator delivered a brief, synchronized rotational thrust with both hands (Fig. 3). The contralateral sitting manipulation was performed in reverse.

All manipulations in both groups were performed by the same experienced clinician to maintain consistency in the contact position, direction of force, and manipulation technique. Each subject underwent three repeated procedures on each side during the same experimental session. These repetitions were performed to obtain repeated measurements and reduce the influence of occasional variability in force acquisition, rather than to represent the usual number of manipulations delivered during routine clinical treatment. During each session, an assistant operated the data acquisition software in real time, and the recorded force data were exported and stored in Excel format for subsequent analysis.

#### Statistical analysis

Statistical analyses of the force data were performed using SPSS version 26.0 (IBM Corp., Armonk, NY, USA). Data with a normal distribution were expressed as mean ± standard deviation ($\:\overline{x}\pm\:s$), whereas non-normally distributed data were described as median with interquartile range (25th–75th percentiles). For left–right comparisons within each manipulation group, paired-sample t-tests were applied when the paired differences met the assumption of normality, while non-normally distributed paired differences were analyzed using the Wilcoxon signed-rank test. Statistical significance was set at *P* < 0.05. In addition, RStudio version 4.4.3 (R Foundation for Statistical Computing, Vienna, Austria) was used to identify the optimal number of clusters via the elbow method and silhouette score. K-means clustering was then performed, yielding the sample size of each cluster and the mean values of feature variables, with results further illustrated by two-dimensional scatter plots and visualization of cluster centers.

### Finite element modeling

A healthy adult female volunteer (25 years old, 165 cm in height, 60 kg in weight) with no history of spinal disease was recruited. The study purpose and potential radiation risks were explained, and written informed consent was obtained. This study was approved by the Medical Ethics Committee of Wangjing Hospital, China Academy of Chinese Medical Sciences (ethics approval No. WJEC-KT-2022-026-P002). CT imaging was performed using a 64-slice spiral CT scanner (SIEMENS, Germany) covering vertebral levels T10 to S5. A total of 261 axial images were acquired with a slice thickness of 0.30 mm, a pitch of 1.25, a matrix size of 512 × 512 pixels, and in-plane resolution of 0.607 × 0.607 mm. All data were stored in Digital Imaging and Communications in Medicine (DICOM) format on optical media.

CT images were first processed using Mimics 21.0 (Materialise, Belgium) to convert two-dimensional data into three-dimensional structures. Bone and surrounding soft tissues were segmented by applying a threshold of 226–1450 Hounsfield units (HU), and each lumbar segment was manually refined using Region Grow, Split Mask, Edit Masks, and Cavity Fill tools before three-dimensional surface reconstruction. The resulting lumbar surface mesh model was exported in Stereolithography (STL) format and imported into Geomagic Wrap 2021 (Geomagic, USA) for optimization and surface reconstruction, yielding a non-uniform rational B-spline (NURBS) surface model. Cancellous bone was generated concurrently, with cortical bone thickness set to 1.5 mm in accordance with previous studies [13, 14]. Both the complete lumbar model and cancellous bone model were exported in Standard for the Exchange of Product model data (STEP; .stp) format. Subsequently, SolidWorks 2021 (Dassault Systèmes, USA) was used for model assembly, interference checking, and further processing, as well as for construction of facet cartilage and intervertebral discs. Following published protocols [15–17], discs were subdivided into nucleus pulposus and annulus fibrosus at a volumetric ratio of 47:53, with superior and inferior endplates (0.25 mm thickness each) incorporated.

**Fig. 4:**
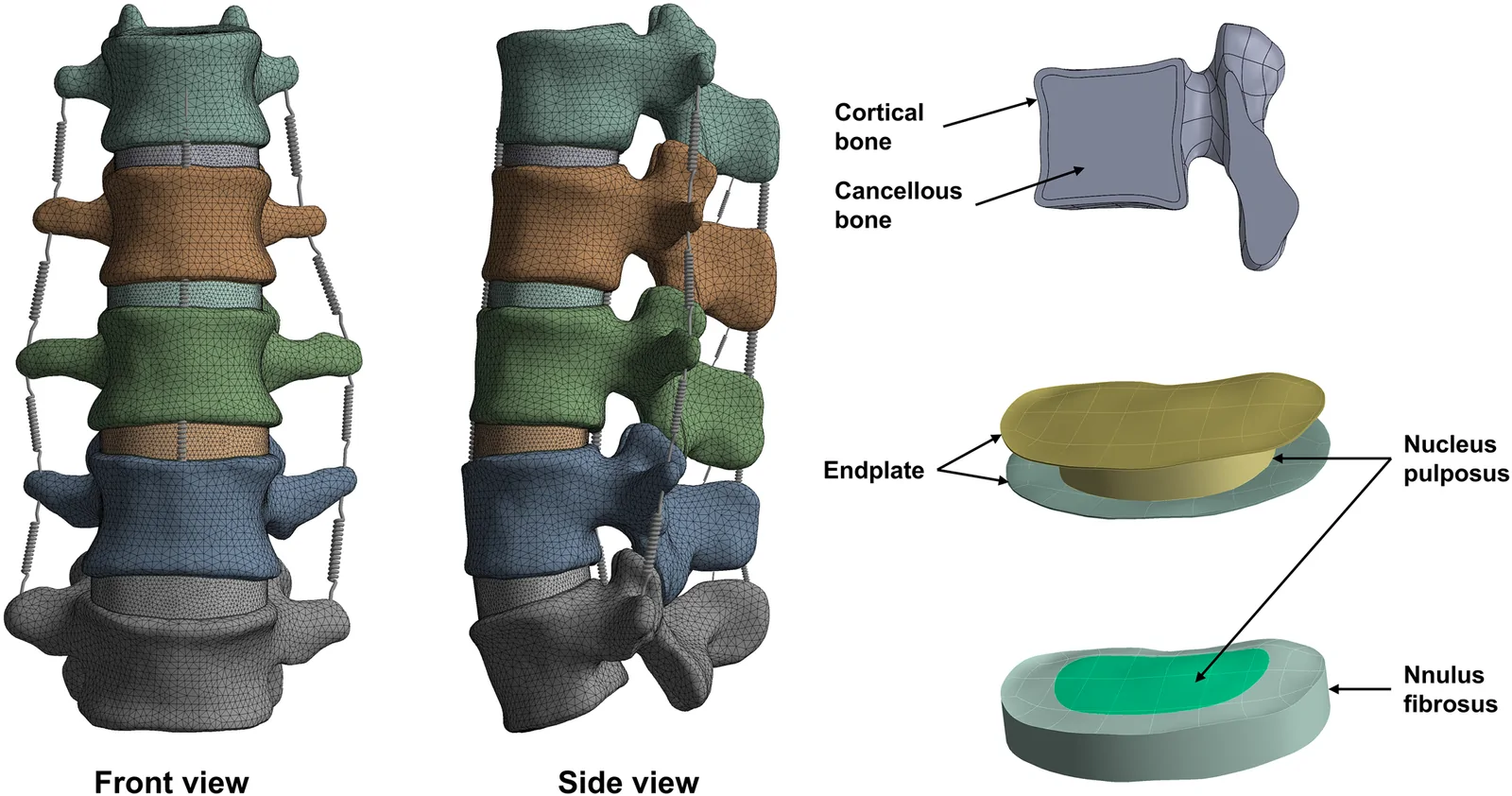
Complete finite element model of the lumbar spine

The complete lumbar solid model was then imported into Ansys Workbench 21.0 (Ansys, USA) in Parasolid text (.x_t) format for finite element analysis. Ligamentous structures—including the anterior and posterior longitudinal ligaments, ligamentum flavum, interspinous ligament, supraspinous ligament, and intertransverse ligament—were represented using spring elements. The solid tissues, including the vertebrae, endplates, annulus fibrosus, nucleus pulposus, and facet cartilage, were modeled as homogeneous, isotropic, linear elastic materials. Material properties for vertebrae, intervertebral discs, cartilage, and ligaments were assigned based on prior literature [18–20] (Table 1). Contact definitions were set as bonded among cortical bone, cancellous bone, annulus fibrosus, nucleus pulposus, and endplates, while facet joints were modeled as no-separation contacts with the facet cartilage of the superior vertebra and as bonded contacts with the inferior vertebra. To balance accuracy and computational efficiency, tetrahedral elements were used for meshing, with nominal edge lengths, representing the approximate length of each edge of the tetrahedral elements, of 2 mm for cortical and cancellous bone, 1 mm for intervertebral discs, and 0.5 mm for facet cartilage. The final finite element model comprised 892,026 nodes and 556,078 elements. The reconstructed lumbar finite element model is illustrated in Fig. 4.

**Table 1:** Material property definitions for the lumbar finite element model

Material	Elastic Modulus (MPa)	Poisson’s Ratio	Stiffness (*N*/mm)
Cortical bone	12,000	0.3	
Cancellous bone	100	0.3	
Vertebral endplate	25	0.25	
Annulus fibrosus	4.2	0.45	
Nucleus pulposus	1	0.49	
Facet cartilage	10.4	0.4	
Anterior longitudinal ligament			8.74
Posterior longitudinal ligament			5.83
Ligamentum flavum			15.38
Interspinous ligament			10.85
Supraspinous ligament			2.39
Intertransverse ligament			0.19

### Model validation

The inferior surface of the L5 vertebra was fixed to restrict motion in all directions as a boundary condition. Physiologically, the lumbar spine bears a load equivalent to approximately two-thirds of total body weight [11, 21]. For the volunteer in this study (60 kg), this corresponded to approximately 400 N, which was applied vertically to the superior surface of L1 to simulate body weight loading. The applied moment magnitude was selected with reference to the in vitro lumbar loading protocol reported by Yamamoto et al. [22], with the superior surface of L1 designated as the loading surface. A moment of 10 N·m was applied in each direction to simulate six motion states: flexion, extension, left and right lateral bending, and left and right axial rotation. The biomechanical responses of the lumbar spine were then computed, and the segmental ranges of motion (ROMs) were compared with the corresponding cadaveric experimental data reported by Yamamoto et al. to assess model validity. An exploratory Bland-Altman analysis was performed separately for each motion category across the four segments. Differences were defined as model predictions minus reference means, and descriptive agreement limits were calculated as the mean difference ± 1.96 standard deviations of the differences.

### Loading conditions

Following validation of the finite element model, the force parameters derived from the clusters identified for each manipulation technique were applied to the model, using the subject’s right side as an example. The loading procedures were as follows:

#### Lateral decubitus lumbar rotational manipulation

For the lateral decubitus manipulation, the right-hand force exerted by the operator on the subject’s shoulder was multiplied by the horizontal distance from the shoulder contact point to the spinal axis (20 cm) to calculate the rotational moment applied to L1. Similarly, the left-hand force applied to the subject’s hip was multiplied by the 20 cm distance from the hip contact point to the spinal axis to determine the counter-rotational moment applied to L5. The calculated moments were then imposed on the L1 and L5 segments of the validated finite element model.

#### Sitting lumbar rotational manipulation

The neutral sitting posture of the subject was first simulated. Under physiological conditions, the lumbar spine supports approximately two-thirds of total body weight, corresponding to an axial compressive load of approximately 400 N applied to the superior surface of L1. On this basis, the right-hand force exerted by the operator on the subject’s shoulder was multiplied by the horizontal distance from the shoulder contact point to the spinal axis (20 cm) to calculate the rotational moment applied to L1. Simultaneously, the maximum force of the operator’s left thumb applied to the subject’s waist was multiplied by the 6 cm distance from the waist contact point to the spinal axis to determine the counter-rotational moment applied to L4. The computed axial load and rotational moments were then imposed on the L1 and L4 segments of the validated finite element model.

## Results

### Force analysis of the two rotational manipulations

A total of 20 healthy volunteers were enrolled and randomly assigned to two groups (*n* = 10 each). Following a standardized experimental protocol, each participant underwent three repeated lumbar rotational manipulations on each side during the same experimental session. After quality control, 120 valid force datasets were collected, comprising 60 datasets for each intervention type.

#### Force analysis of the lateral decubitus lumbar rotational manipulation

For the 10 participants who underwent the lateral decubitus lumbar rotational manipulation, shoulder and hip force parameters were analyzed with emphasis on left-right differences. No statistically significant differences in these force parameters were observed between the left and right sides (all *P* > 0.05), suggesting that the kinematic parameters of the lateral decubitus manipulation were comparable regardless of laterality. Detailed results are presented in Table 2.

**Table 2:** Statistical analysis of force parameters for the lateral decubitus lumbar rotational manipulation

Force parameter	Left side (*n* = 30)	Right side (*n* = 30)	t / Z statistic	*P*-value
Shoulder force (N)	51.99 ± 15.66	57.00 ± 17.42	-1.231	0.228
Hip force (N)	85.60(72.45–103.66)	81.48(60.17–124.53)	-0.946	0.344

#### Cluster analysis of force parameters for the lateral decubitus lumbar rotational manipulation

**Fig. 5:**
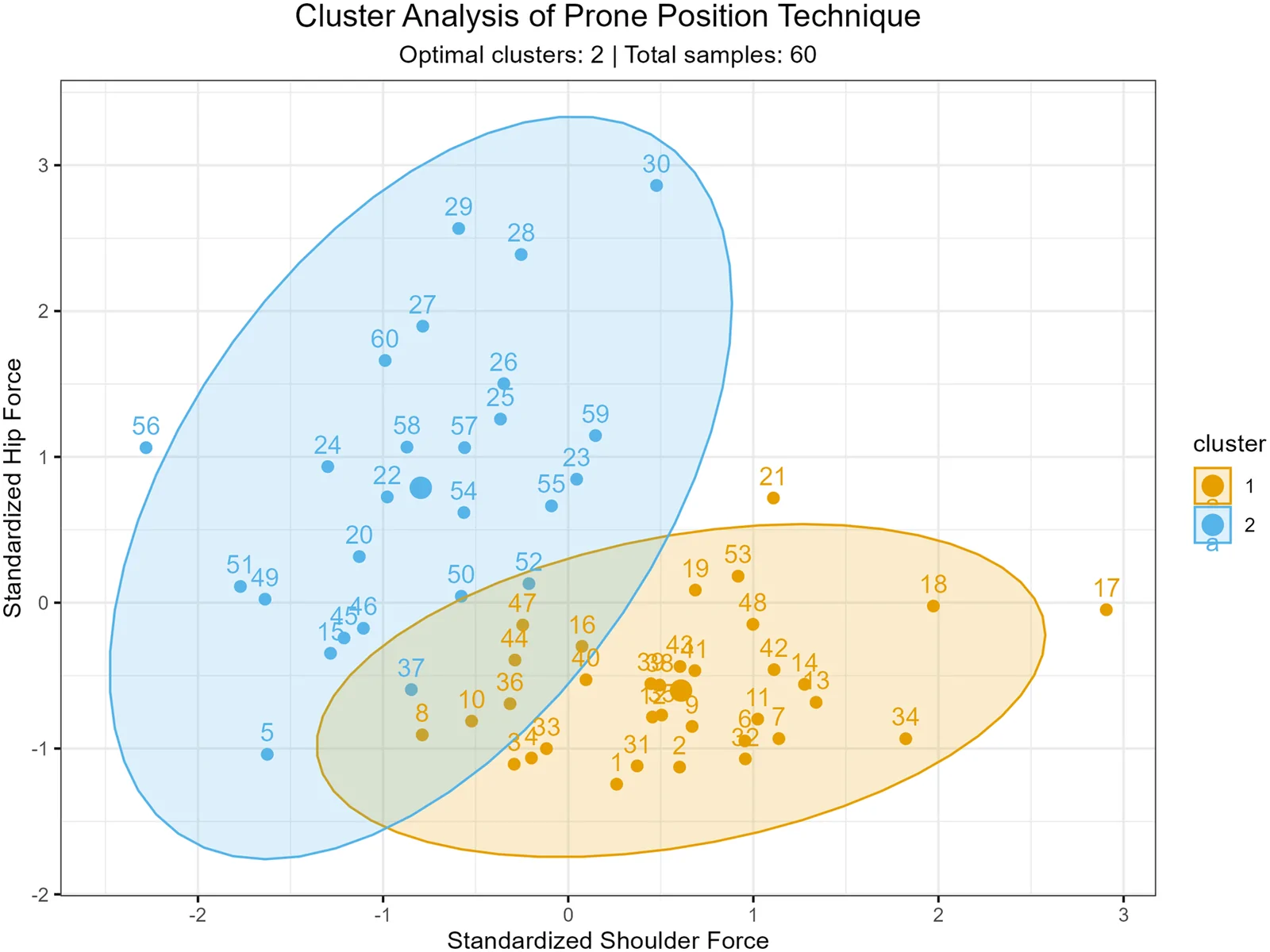
Cluster analysis of force parameters for the lateral decubitus lumbar rotational manipulation

The optimal number of clusters was identified as *k* = 2 based on the elbow method and silhouette score. K-means clustering of the force parameters yielded two distinct operational patterns (Table 3; Fig. 5):

Cluster 1 (shoulder-hip coordinated pattern): Shoulder and hip forces were comparable (shoulder: 64.61 N; hip: 71.37 N), indicating that both regions contributed synergistically to the manipulation, resulting in a balanced distribution of loading.

Cluster 2 (hip-dominant pattern): Shoulder force was relatively low (41.26 N), whereas hip force was substantially higher (121.62 N). This mode was characterized by hip-dominated loading, with the shoulder playing only a supporting role.

**Table 3:** Cluster analysis of force parameters for the lateral decubitus lumbar rotational manipulation

Cluster	Shoulder mean force (*N*)	Hip mean force (*N*)	Number of samples (*n*)	Percentage
Cluster 1	64.61	71.37	34	56.7%
Cluster 2	41.26	121.62	26	43.3%

#### Force analysis of the sitting lumbar rotational manipulation

For the 10 participants undergoing the sitting lumbar rotational manipulation, shoulder and waist loading parameters were analyzed with emphasis on left-right differences. Significant side-to-side differences were observed (*P* < 0.05): shoulder loading was lower on the left than on the right, whereas waist loading was higher on the left compared with the right. Detailed results are presented in Table 4.

**Table 4:** Statistical analysis of force parameters for the sitting lumbar rotational manipulation

Force parameter	Left side (*n* = 30)	Right side (*n* = 30)	t / Z statistic	*P*-value
Shoulder force (N)	62.56 ± 15.58	77.70 ± 7.72	-10.088	< 0.001
Waist force (N)	32.80(27.75–38.20)	26.06(22.66–33.54)	2.129	0.042

#### Cluster analysis results of force parameters of the sitting lumbar rotational manipulation

**Fig. 6:**
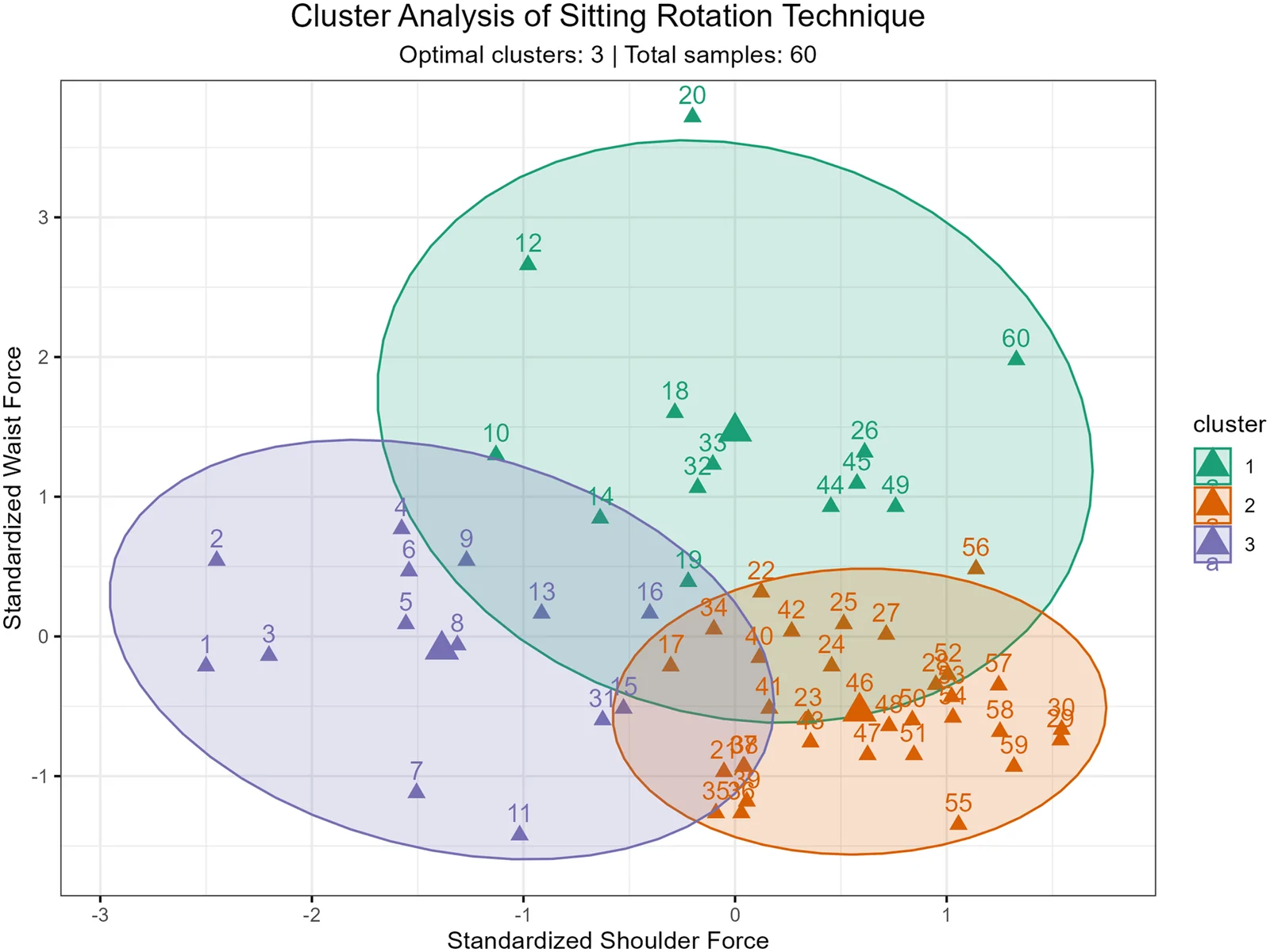
Cluster analysis of force parameters for the sitting lumbar rotational manipulation

The optimal number of clusters was determined as *k* = 3 using the elbow method and silhouette score. K-means clustering was then applied to the obtained force parameters, and the results are shown in Table 5; Fig. 6. According to the clustering results, the sitting lumbar rotational manipulation can be divided into three clusters:

Cluster 1 (shoulder-waist coordinated pattern): Shoulder force was moderate (70.13 N), waist force was highest (47.37 N), and the manipulation was performed with coordinated force exertion by the shoulder and waist.

Cluster 2 (shoulder-dominant pattern): Shoulder force was highest (78.59 N), waist force was lowest (26.16 N), and the manipulation was dominated by shoulder force, with the waist acting only as an auxiliary. This type accounted for the largest proportion, 55.0%.

Cluster 3 (gentle-loading pattern): Shoulder force was lowest (50.20 N), waist force was moderate (30.85 N), and the manipulation involved relatively small overall loading, possibly corresponding to smaller-bodied subjects.

**Table 5:** Cluster analysis of force parameters for the sitting lumbar rotational manipulation

Cluster	Shoulder mean force (*N*)	Waist mean force (*N*)	Number of samples (*n*)	Percentage
Cluster 1	70.13	47.37	13	21.7%
Cluster 2	78.59	26.16	33	55.0%
Cluster 3	50.20	30.85	14	23.3%

### Finite element model validation results

Validation results for segmental ROM in flexion-extension, lateral bending, and axial rotation are summarized in Table 6. The findings demonstrated broadly comparable ROM magnitudes between the present results and the previously reported cadaveric data [22]. Exploratory Bland-Altman analysis showed mean model-minus-reference differences of -0.125°, -1.575°, and 0.525° for flexion-extension, lateral bending, and axial rotation, respectively, with corresponding descriptive agreement limits of -2.350° to 2.100°, -4.240° to 1.090°, and − 0.553° to 1.603°. These results supported the biomechanical plausibility of the finite element model for subsequent manipulative loading simulations and biomechanical analyses.

**Table 6:** Validation of segmental range of motion in flexion-extension, lateral bending, and axial rotation for the lumbar finite element model

Present study	Flexion-Extension (°)	Left-Right Lateral Bending (°)	Left-Right Axial Rotation (°)
L1/2	10.1	9.9	5.5
L2/3	11.0	10.7	6.2
L3/4	12.2	10.2	5.6
L4/5	13.0	9.7	4.2
Yamamoto in vitro study	Flexion-Extension (°)	Left-Right Lateral Bending (°)	Left-Right Axial Rotation (°)
L1/2	10.1 ± 1.1	9.9 ± 0.8	4.6 ± 1.1
L2/3	10.8 ± 0.6	14.0 ± 1.2	5.2 ± 0.8
L3/4	11.2 ± 1.1	11.5 ± 0.8	5.2 ± 0.8
L4/5	14.7 ± 1.1	11.4 ± 1.0	4.4 ± 0.8

### Finite element model loading results

#### Loading outcomes under different conditions

Lateral decubitus lumbar rotational manipulation: (1) Shoulder-hip coordinated pattern: A right rotational moment of 12.922 N·m was applied to L1, and a left rotational moment of 14.274 N·m to L5. (2) Hip-dominant pattern: A right rotational moment of 8.252 N·m was applied to L1, and a left rotational moment of 24.324 N·m to L5.

Sitting lumbar rotational manipulation: An axial compressive load of 400 N was applied to the superior surface of L1. On this basis: (1) Shoulder-waist coordinated pattern: A right rotational moment of 14.026 N·m was applied to L1, and a left rotational moment of 2.842 N·m to L4. (2) Shoulder-dominant pattern: A right rotational moment of 15.718 N·m was applied to L1, and a left rotational moment of 1.570 N·m to L4. (3) Gentle-loading pattern: A right rotational moment of 10.040 N·m was applied to L1, and a left rotational moment of 1.851 N·m to L4.

#### Finite element analysis results

Finite element modeling was employed to evaluate the biomechanical effects of five clusters derived from the lateral decubitus and sitting lumbar rotational manipulations. Particular attention was given to stress and displacement distributions within the intervertebral discs and facet cartilage. The findings are summarized below:

##### Lateral decubitus lumbar rotational manipulation

**Fig. 7:**
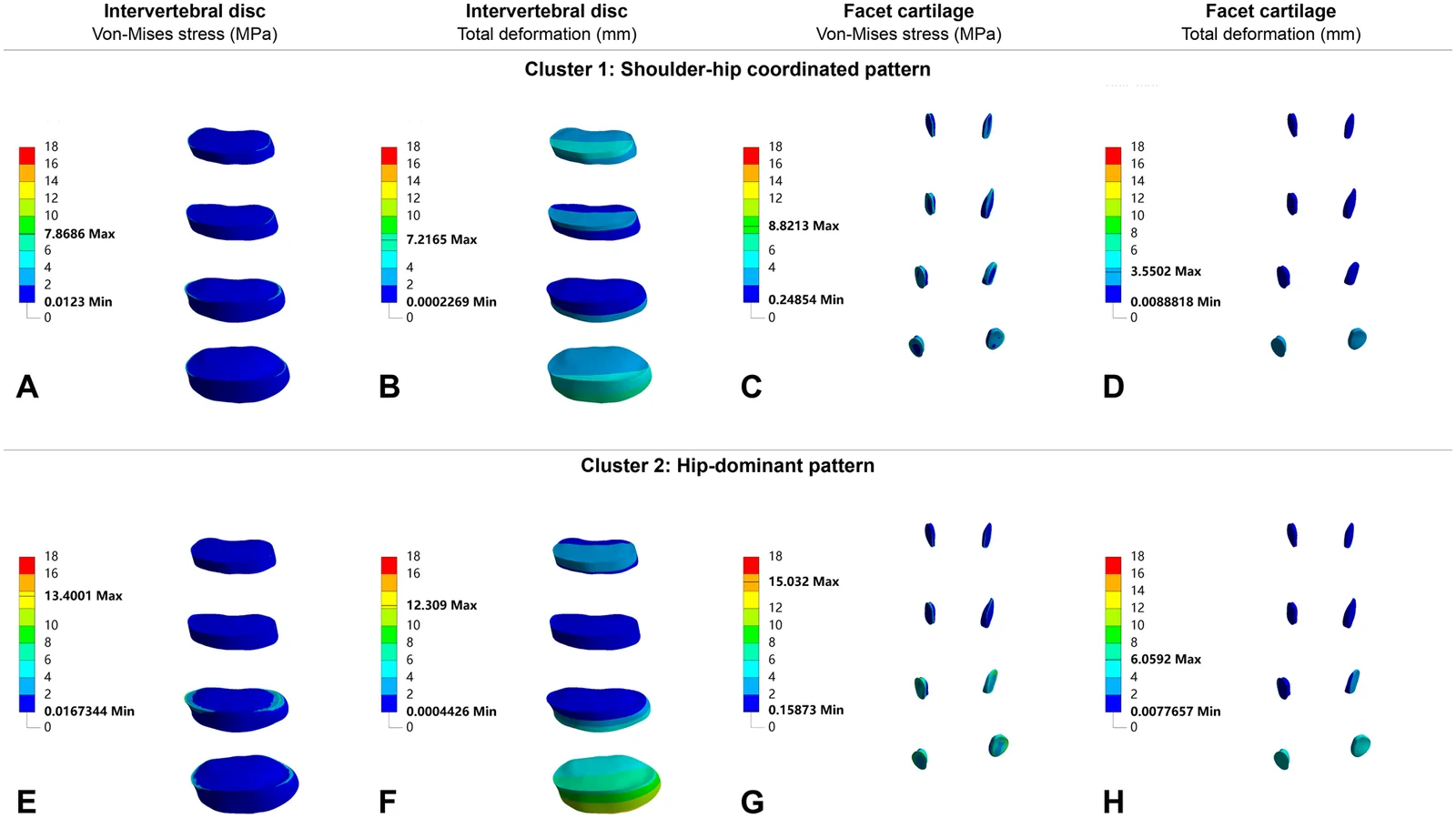
Finite element analysis of stress and displacement in two clusters of the lateral decubitus lumbar rotational manipulation. (**A**) Disc von-Mises stress (MPa) in the shoulder-hip coordinated pattern; (**B**) Disc total deformation (mm) in the shoulder-hip coordinated pattern; (**C**) Facet cartilage von-Mises stress (MPa) in the shoulder-hip coordinated pattern; (**D**) Facet cartilage total deformation (mm) in the shoulder-hip coordinated pattern; (**E**) Disc von-Mises stress (MPa) in the hip-dominant pattern; (**F**) Disc total deformation (mm) in the hip-dominant pattern; (**G**) Facet cartilage von-Mises stress (MPa) in the hip-dominant pattern; (**H**) Facet cartilage total deformation (mm) in the hip-dominant pattern

In both the shoulder-hip coordinated and hip-dominant patterns, the distribution of peak stress and displacement in the intervertebral discs and facet cartilage was largely consistent. Peak von-Mises stress of the disc occurred at L3/4, while maximum total deformation was observed at L4/5. For facet cartilage, peak von-Mises stress was localized to the left side of L4/5, whereas maximum total deformation appeared on the right side of L4/5. The shoulder-hip coordinated pattern exhibited moderate stress and displacement with relatively uniform distribution. By contrast, the hip-dominant pattern produced higher stress and displacement values in the discs and cartilage at L3/4 and L4/5, but lower values at L1/2 and L2/3, indicating a more concentrated mechanical effect in the lower lumbar region. Detailed finite element results are illustrated in Fig. 7.

##### Sitting lumbar rotational manipulation

**Fig. 8:**
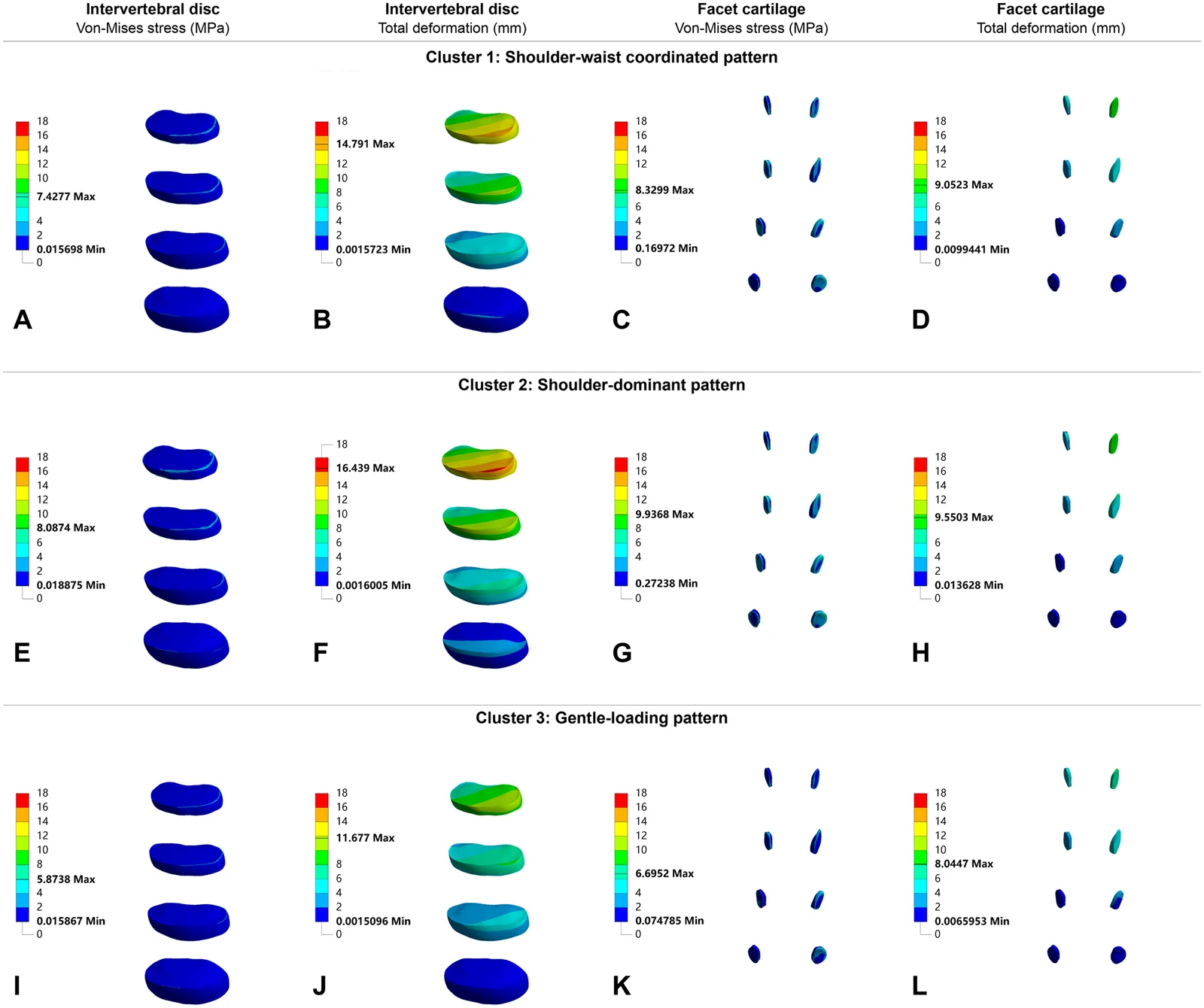
Finite element analysis of stress and displacement in three clusters of the sitting lumbar rotational manipulation. (**A**) Disc von-Mises stress (MPa) in the shoulder-waist coordinated pattern; (**B**) Disc total deformation (mm) in the shoulder-waist coordinated pattern; (**C**) Facet cartilage von-Mises stress (MPa) in the shoulder-waist coordinated pattern; (**D**) Facet cartilage total deformation (mm) in the shoulder-waist coordinated pattern; (**E**) Disc von-Mises stress (MPa) in the shoulder-dominant pattern; (**F**) Disc total deformation (mm) in the shoulder-dominant pattern; (**G**) Facet cartilage von-Mises stress (MPa) in the shoulder-dominant pattern; (**H**) Facet cartilage total deformation (mm) in the shoulder-dominant pattern; (**I**) Disc von-Mises stress (MPa) in the gentle-loading pattern; (**J**) Disc total deformation (mm) in the gentle-loading pattern; (**K**) Facet cartilage von-Mises stress (MPa) in the gentle-loading pattern; (**L**) Facet cartilage total deformation (mm) in the gentle-loading pattern

In the shoulder-waist coordinated, shoulder-dominant, and gentle-loading patterns of the sitting lumbar rotational manipulation, the locations of peak stress and displacement in the intervertebral discs and facet cartilage were identical. Peak disc von-Mises stress occurred at L2/3, while maximum total deformation was observed at L1/2. For facet cartilage, peak von-Mises stress was localized to the left side of L4/5, and peak total deformation to the left side of L1/2. Although the three clusters shared a similar mechanical distribution pattern, the shoulder-dominant pattern consistently produced greater stress and displacement across segments, particularly at L1/2 and L2/3, indicating more pronounced tensile and compressive responses. In contrast, the gentle-loading pattern demonstrated substantially lower stress and displacement at the same levels, with milder disc responses and relatively weaker stress concentration and deformation in the facet cartilage, reflecting a more conservative mechanical profile. Detailed finite element results are illustrated in Fig. 8.

**Fig. 9:**
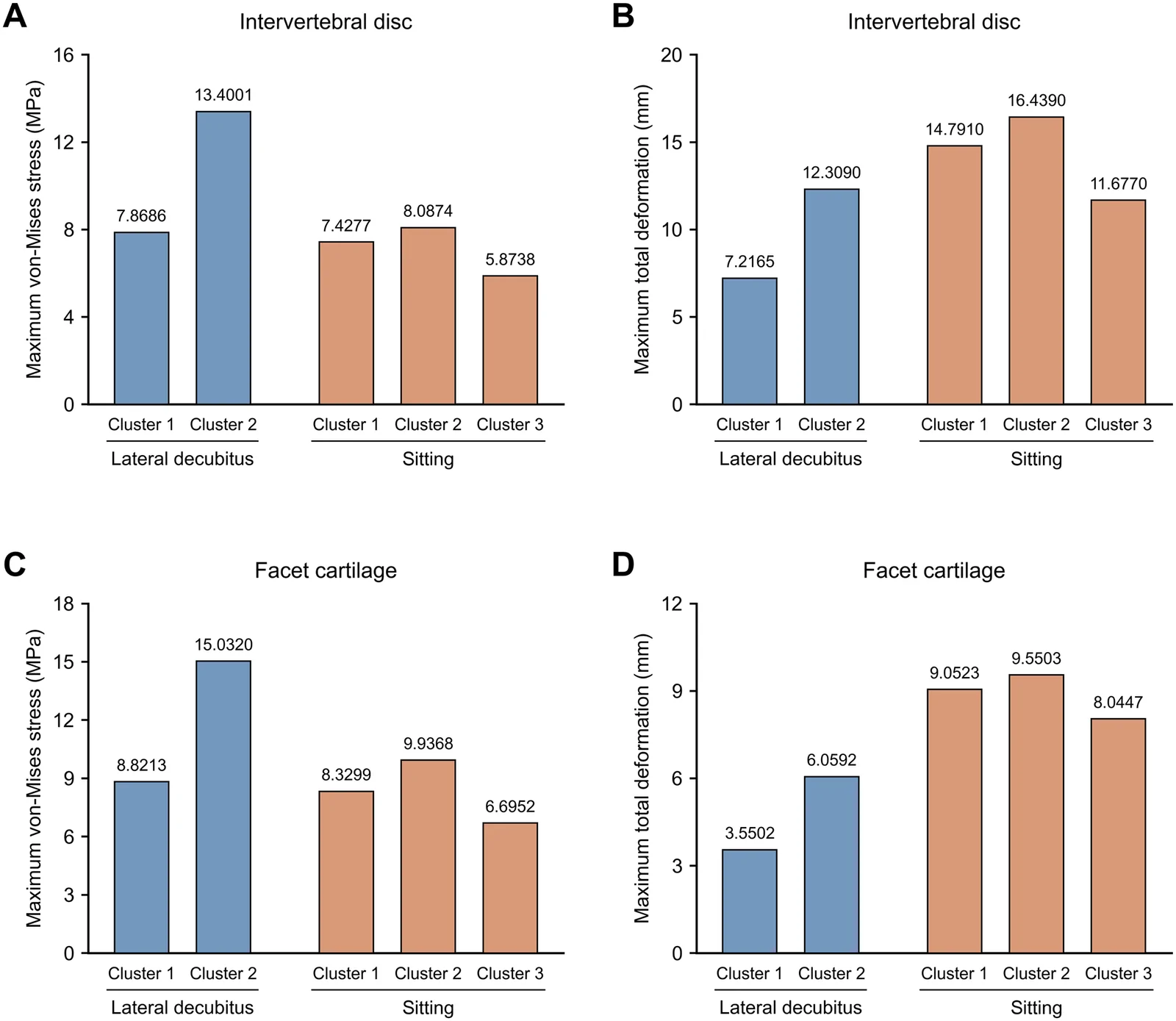
Finite element stress and displacement comparison for five clusters of the two lumbar rotational manipulations. (**A**) Peak von-Mises stress (MPa) of the intervertebral disc; (**B**) Maximum total deformation (mm) of the intervertebral disc; (**C**) Peak von-Mises stress (MPa) of the facet cartilage; (**D**) Maximum total deformation (mm) of the facet cartilage

Finite element analysis revealed distinct differences in stress and displacement patterns between the lateral decubitus and sitting lumbar rotational manipulations. The lateral decubitus manipulation generally generated higher peak stresses but lower peak displacements in the intervertebral discs and facet cartilage, reflecting a high-stress/low-displacement profile. Both clusters primarily affected the L3/4 and L4/5 segments, with the lower lumbar region subjected to greater loading, particularly in the hip-dominant pattern where stresses were markedly elevated. In contrast, the sitting manipulation generally exhibited lower stress peaks but substantially higher displacement peaks, consistent with a low-stress/high-displacement pattern. Its three clusters mainly influenced the upper lumbar region (L1/2 and L2/3), accompanied by stress concentration at the L4/5 facet joints. Within these, the shoulder-dominant pattern demonstrated the strongest mechanical effects, whereas the gentle-loading pattern produced the lowest peak stress and displacement values, reflecting a milder biomechanical impact. Statistical results of stress and displacement across the five clusters of both manipulations are summarized in Table 7; Fig. 9.

**Table 7:** Finite element stress and displacement statistics for five clusters of the two lumbar rotational manipulations

		Lateral decubitus manipulation		Sitting manipulation		
		Shoulder-hip coordinated pattern	Hip-dominant pattern	Shoulder-waist coordinated pattern	Shoulder-dominant pattern	Gentle-loading pattern
Von-Mises stress of the intervertebral disc (MPa)	Max	7.8686	13.4001	7.4277	8.0874	5.8738
	Average	0.6743	0.7720	0.7623	0.8470	0.5985
	Min	0.0123	0.0167	0.0157	0.0189	0.0159
Total deformation of the intervertebral disc (mm)	Max	7.2165	12.3090	14.7910	16.4390	11.6770
	Average	2.4436	3.2221	4.9275	5.5405	3.9523
	Min	0.0002	0.0004	0.0016	0.0016	0.0015
Von-Mises stress of the facet cartilage (MPa)	Max	8.8213	15.0320	8.3299	9.9368	6.6952
	Average	2.7731	3.4454	2.6842	3.0998	1.9414
	Min	0.2485	0.1587	0.1697	0.2724	0.0748
Total deformation of the facet cartilage (mm)	Max	3.5502	6.0592	9.0523	9.5503	8.0447
	Average	1.3787	1.9990	2.9286	3.0370	2.7382
	Min	0.0089	0.0078	0.0099	0.0136	0.0066

## Discussion

This study integrated in vivo biomechanical measurements with in silico finite element analysis to evaluate the characteristics of practitioner-generated forces during lumbar rotational manipulation and the resulting mechanical responses of lumbar tissues. Using instrumented gloves, the peak forces generated during manipulation were recorded and incorporated as boundary conditions into a high-fidelity three-dimensional L1–L5 finite element model. This approach enabled simulation of the immediate biomechanical responses associated with five clusters of lateral decubitus and sitting lumbar rotational manipulations. The findings provide insight into stress-displacement distributions and segmental specificity of the two techniques, offering a biomechanical rationale to support more precise clinical application.

### Lateral decubitus manipulation demonstrates greater force symmetry than sitting manipulation

Using instrumented gloves, this study quantified the manipulative forces applied to 20 volunteers and revealed fundamental differences in loading characteristics between lateral decubitus and sitting lumbar rotational manipulations. The lateral decubitus technique exhibited a bilaterally symmetric force pattern, with both hands contributing to the maneuver. Force application showed minimal asymmetry (< 10% deviation, *P* > 0.05), indicating relatively consistent loading between the left and right sides. Its predominant cluster was the shoulder-hip coordinated pattern (56.7%). In contrast, the sitting manipulation displayed a markedly asymmetric, shoulder-dominant mode: the shoulder hand accounted for over 65% of the applied force, while the waist hand served a supporting role, resulting in significant side-to-side variation (> 20% deviation, shoulder: *P* < 0.001; waist: *P* = 0.042). Its dominant cluster was the shoulder-dominant pattern (55.0%). The mean contact forces across the identified manipulation clusters ranged from 26.16 to 121.62 N. These values were comparable in magnitude to the preload force of 88 N reported by Herzog for sacroiliac joint manipulation, but were substantially lower than the reported peak thrust force of 323 N [23]. This difference may be related to variations in manipulation technique, contact site, loading phase, force-measurement method, and practitioner characteristics.

Previous studies have shown that manipulative force-time characteristics may vary across spinal regions and among practitioners [24]. Colloca et al. further examined thrusts delivered with the left and right hands and reported that handedness, training, and clinical experience may influence biomechanical performance during manipulation [25]. These practitioner-related factors may provide some context for the greater side-to-side variation observed during sitting manipulation. However, because all procedures in the present study were performed by a single clinician, the effect of hand dominance cannot be separated from the clinician’s individual technical characteristics. These operational differences may contribute to distinct biomechanical characteristics: the lateral decubitus manipulation, through coordinated bilateral loading, may promote more consistent force transmission to the lower lumbar segments, whereas the sitting manipulation, driven predominantly by shoulder loading, enhances upper lumbar displacement but may be more susceptible to side-to-side variability. Clinically, this underscores the importance of standardizing contact position, force application, and bilateral operational consistency to ensure safe and effective practice.

### Opposing biomechanical trends between lateral decubitus and sitting manipulations identified by finite element analysis

Finite element simulations incorporating clustered force parameters from the five clusters revealed contrasting biomechanical behaviors between the two manipulations. The lateral decubitus manipulation displayed a high-stress/low-displacement profile, with peak disc stress reaching 13.40 MPa at L3/4 and maximum displacement of 12.31 mm at L4/5. Conversely, the sitting manipulation exhibited a low-stress/high-displacement pattern: disc and cartilage stresses were generally lower than those in the lateral decubitus technique, but upper lumbar segments–particularly L1/2–showed markedly greater displacement.

These divergent patterns can be explained by distinct load transmission pathways. In the lateral decubitus manipulation, rigid pelvic loading created a short lever arm, channeling stress through the sacroiliac joint directly to the lower lumbar region (L3–S1). This focused transmission produced stress and displacement peaks at the L3/4 disc and L4/5 facet cartilage. In contrast, the sitting manipulation relied on axial spinal rotation with a long lever-arm effect. Reaching the end range required greater displacement, with peaks localized in the upper lumbar spine. Because forces were distributed over longer spinal segments, stress magnitudes in the discs and facet cartilage were substantially reduced.

These findings corroborate the “proximal concentration effect” of spinal load transfer, whereby segments closer to the point of force application experience greater stress concentration [26, 27]. Thus, lateral decubitus manipulation, involving simultaneous shoulder and pelvic loading, concentrated effects in the lower lumbar spine, while the shoulder-dominant sitting manipulation shifted stress and displacement to the proximal upper lumbar segments.

### Clinical and safety implications: lateral decubitus manipulation for targeted high-stress release of the lower lumbar spine, sitting manipulation for high-displacement expansion of the upper lumbar spine

Given the contrasting biomechanical profiles of lateral decubitus and sitting lumbar rotational manipulations, their clinical indications and risk management should be clearly distinguished. From a clinical decision-making perspective, lateral decubitus manipulation may be more suitable for lower lumbar lesions requiring localized release, whereas sitting manipulation may be more appropriate for upper lumbar conditions in which greater decompressive displacement is desired. For lower lumbar pathologies, such as L4/5 facet dysfunction or lumbosacral fascial strain, the lateral decubitus technique concentrates stress at the L3/4 disc and L4/5 facet cartilage, enabling effective release of adhesions and stiffness in the lower lumbar region. However, the predicted peak stresses were of a similar magnitude to the reported yield strength of cancellous bone (~ 10 MPa) [28], suggesting that caution is warranted in patients with poor bony tolerance, endplate vulnerability, or marked degeneration. Nevertheless, the calculated von-Mises stresses cannot be directly equated with tissue failure loads because of differences in the evaluated mechanical parameters, tissue types, and loading conditions. Moreover, no tissue-specific failure criterion was incorporated into the present linear elastic model; therefore, a definitive safety factor could not be determined.

For upper lumbar indications, including thoracolumbar junction stiffness or high-level nerve root compression, the sitting manipulation induces pronounced displacement at the L1/2 disc and facet cartilage. This facilitates expansion of the upper intervertebral foramina and spinal canal, thereby alleviating neural compression. Nonetheless, excessive displacement may increase the risk of traction-related nerve injury, highlighting the need for precise control of manipulation amplitude [29, 30]. Clinically, this technique may therefore be preferable when decompressive motion is needed at the upper lumbar segments, but special attention should be paid to displacement magnitude and neurological sensitivity.

Cluster-specific variations also have important clinical safety implications. In the lateral decubitus technique, the hip-dominant pattern produced L4/5 disc displacement of up to 12.31 mm, exceeding the normal physiological range and therefore warranting caution in patients undergoing disc repair [31]. In the sitting technique, the shoulder-dominant pattern generated the highest stress at L2/3, although the absolute stress level remained relatively low; particular caution may nevertheless be required in patients with osteoporosis [32, 33]. Importantly, both manipulations consistently produced stress concentration in the L4/5 facet cartilage, indicating that this region deserves particular clinical attention. Overall, these findings suggest that the two manipulations exhibit distinct biomechanical safety profiles: lateral decubitus manipulation is characterized by greater stress-related loading in the lower lumbar spine, whereas sitting manipulation is more closely associated with displacement-related effects in the upper lumbar region. These results should be interpreted as relative stress and displacement patterns rather than as direct evidence of tissue failure or absolute clinical safety.

### Limitations

This study has several limitations. First, force data were collected exclusively from healthy young adults with a limited sample size, without stratification by age or BMI, which restricts the generalizability of the findings to broader or at-risk populations. In particular, the sample size was relatively small for cluster analysis; therefore, the identified clusters should be regarded as preliminary exploratory patterns requiring validation in larger cohorts. All manipulations were performed by a single clinician, which improved procedural consistency but may limit generalizability to other practitioners. Moreover, three repetitions were performed on each side during the same session, and the later recordings may have been influenced by joint gapping, tissue relaxation, or other transient physiological changes caused by the preceding procedures.

Second, the finite element model was based on healthy anatomy and did not incorporate pathological features of lumbar disc herniation (LDH). While sufficient to clarify the fundamental mechanical effects of the two manipulations on intervertebral discs and facet cartilage, this approach lacks disease-specific relevance. The solid tissues were modeled using homogeneous, isotropic, linear elastic properties, which do not fully represent the nonlinear and time-dependent behavior of the annulus fibrosus, nucleus pulposus, and facet cartilage. Facet cartilage was also represented as a single continuous component, which may have introduced some uncertainty in local contact and stress predictions. Third, simulations were performed under idealized conditions without inclusion of lumbar musculature, likely underestimating the complexity of in vivo stress distributions. Finally, static loading was applied, capturing only peak forces without accounting for the temporal profile or acceleration effects of the thrust phase.

### Future directions

Future studies should establish a larger, stratified force database that includes participants across different age and BMI groups, as well as multiple practitioners, to clarify how anatomical diversity and practitioner-related variability affect force transmission. The preliminary manipulation clusters identified in this study also require validation in independent cohorts. Patient-specific finite element models incorporating LDH-related changes, such as reduced disc hydration and graded annular fissures, may help characterize biomechanical responses across different stages of degeneration and further clarify the effects of manipulation on herniated discs and adjacent neural structures. In addition, dynamic in silico simulations that incorporate lumbar muscle loading, viscoelastic and rate-dependent material properties, and time-varying thrust profiles may provide a more physiologically realistic representation of tissue responses during manipulation. Ultimately, these advances may support individualized biomechanical assessment, real-time monitoring of manipulative loading, and more effective risk management in vulnerable populations.

## Conclusion

This study integrated force measurement with finite element analysis to compare the biomechanical profiles of lateral decubitus and sitting lumbar rotational manipulations. The lateral decubitus technique demonstrated a high-stress/low-displacement pattern, with stress concentrated in the lower lumbar spine. By contrast, the sitting manipulation exhibited a low-stress/high-displacement profile, with greater displacement primarily involving the upper lumbar spine. These findings indicate that the two techniques produce distinct regional loading and displacement patterns. Clinically, technique selection should be individualized according to patient characteristics and lesion location, with careful control of force amplitude and bilateral symmetry to minimize the risk of manipulation-related complications.

## Data Availability

The datasets generated and/or analysed during the current study are not publicly available due privacy of the volunteer but are available from the corresponding author on reasonable request.
